# Tumor necrosis factor-alpha promoter polymorphism 308 G/A is not significantly associated with esophageal cancer risk: a meta-analysis

**DOI:** 10.18632/oncotarget.13093

**Published:** 2016-11-04

**Authors:** Ming Luo, Yuan Yang, Dongmei Luo, Liang Liu, Yuening Zhang, Feifan Xiao, Jingcheng Yang, Chengdong Zhang, Shen Fu, Zhiguo Luo

**Affiliations:** ^1^ Department of Clinical Oncology, Taihe Hospital, Hubei University of Medicine, Shiyan, Hubei, China; ^2^ School of Life Sciences, Fudan University, Shanghai, China; ^3^ School of Mathematics and Physics, Anhui University of technology, Maanshan, Anhui, China; ^4^ Department of Oncology, Fudan University Shanghai Cancer Center, Fudan University, Shanghai, China; ^5^ Medical Scientific Research Center, Guangxi Medical University, Nanning, Guangxi, China; ^6^ Department of Radiation Oncology, Shanghai Proton and Heavy Ion Center, Shanghai, China; ^7^ Department of Radiation Oncology, Fudan University Shanghai Cancer Center, Shanghai, China

**Keywords:** esophageal cancer, TNF-α-308 G/A, meta-analysis, risk, association

## Abstract

Many studies have investigated the association between Tumor necrosis factor-α-308 G>A (rs1800629) and the risk of esophageal cancer. However, their results are inconsistent. Therefore, we performed a meta-analysis of available data to investigate any possible association between this polymorphism and esophageal cancer risk. We searched PubMed, EMBASE, Web of Science, and the CNKI database for articles published up to 2016. Crude and adjusted odds ratio with 95% confidence intervals were calculated using fixed or random effects models. We used a dominant model (GA+AA vs GG), a recessive model (AA vs GG+GA), an over-dominant model (GG+AA vs GA), and allele frequency (G vs A) to identify any association. Eleven studies with 5617 participants were included in the meta-analysis. Our results suggest that TNF-α-308 G>A (rs1800629) is not significantly associated with a risk of esophageal squamous cell carcinoma and esophageal adenocarcinoma. For genetic association studies, negative results of meta-analysis have a high level of evidence, and these results are important in this era of high-throughput sequencing-based precision medicine.

## INTRODUCTION

Esophageal cancer (EC) which is the most common tumor of the digestive system has two major subtype: esophageal squamous cell carcinoma (ESCC) and esophageal adenocarcinoma (EAC) [1, 2]. The survival rate of esophageal cancer is < 10% after five years [3], mainly because of its extremely aggressive nature and poor survival [4]. In 2016, an estimated 16,910 new esophageal cancer cases and 15,690 deaths occurred in United States [5].

Various factors can lead to EC: race, smoking, alcohol consumption, diet, nutrients, obesity and genetics [4]. However, we are still not familiar with the Genetic epidemiology. Some Genome-Wide Association Studies (GWAS) have shown that some single nucleotide polymorphisms (SNPs) are risk factors of EC [1, 6, 7].

Tumor necrosis factor α, as a central regular of inflammation, is a powerful pro-inflammatory cytokine [8, 9]. Single nucleotide variation at 308 position of Tumor necrosis factor alpha (TNF α, also known as TNF) has been proved to be altering the expression of TNF in transcriptional level [10, 11]. Abnormal in the expression of TNF has been identified to be associated with major depression, Alzheimer's disease, psoriasis, inflammatory bowel disease and cancer [12–16].

Recently, some studies have focused on the association between TNF-α-308 G>A (rs1800629) and risk of EC. In 2003, Emad et al. found rs1800629 was not associated with risk of ESCC and EAC [17]. In 2010, Oh et al. did not observe association between rs1800629 and risk of ESCC and EAC [18], which was consistent with the findings of David et al [19]. In 2010, Zhang et al. and Zhao et al. suggested that there was no significantly association in ESCC [20, 21]. Cui et al. found no association either in 2015 [22]. However, Umar et al. and Wang et al. showed association in 2013 and 2014 respectively [23, 24]. Due to the inclusive of the results. Here, we conducted a meta-analysis based on previously published studies to get a comprehensive result.

## RESULTS

### Literature search

**Figure 1:**
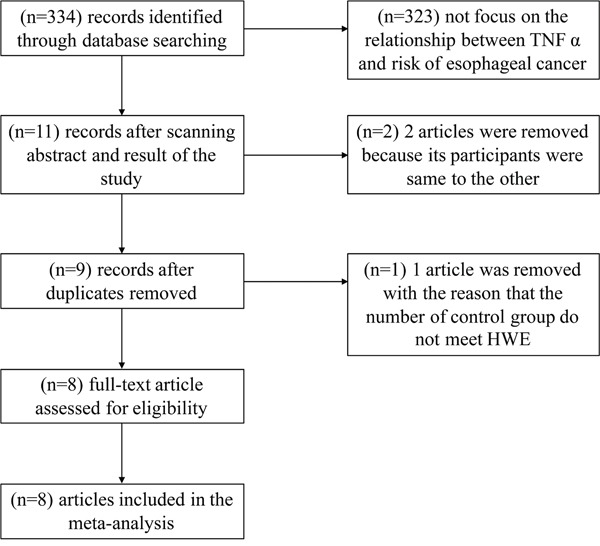
A flow diagram for the study selection process In all, 334 articles were identified using the search strategy. Of these, 323 articles were excluded because they do not investigate the association between rs1800629 and risk of esophageal cancer and 11 articles were screened further. Of the 11 articles,1 article was excluded because the genotype of the case-control group did not satisfy the Hardy-Weinberg Equilibrium (HWE), and 2 articles were excluded with the reason that their participants were same to the other. Finally, 8 publications with 11 case-control studies were included in the meta-analysis.

A flow diagram for the study selection process is shown in Figure 1. In all, 334 articles were identified using the search strategy. Of these, 323 articles were excluded because they do not investigate the association between rs1800629 and risk of esophageal cancer and 11 articles were screened further. Of the 11 articles, 1 article was excluded because the genotype of the case-control group did not satisfy the Hardy-Weinberg Equilibrium (HWE), and 2 articles were excluded with the reason that their participants were same to the other. Finally, 8 publications with 11 case-control studies were included in the meta-analysis. The 8 publications comprised 6 English-language papers and 2 Chinese-language papers.

### Study characteristics

The primary characteristics of the 11 studies are summarized in Table 1. In all, 5617 participants (1855 cases and 3762 controls) were included in the meta-analysis. The studies were divided into 2 groups based on the type of esophageal cancer: 8 case-control studies involving ESCC and 3 case-control studies involving EAC. In order to reduce the influences of ethnicity on the result, we did subgroup analysis according to the ethnicity of their participants.

**Table 1:** Characteristics of the 8 studies included in the meta-analysis

Author	Year	Country	Ethnicity	Type^a^	Cases			Controls			*P* value of HWE^b^	Susceptibility^c^	Adjusted factors
					GG	GA	AA	GG	GA	AA			
**Emad *et al.***	2003	USA	Mix	ESCC	41	10	2	152	52	6	0.54823	N	Age, sex, BMI, smoking, alcohol, ethnicity, history of reflux symptoms, history of peptic ulcer, family history of cancer, lauren classification, DNA source, and H. pylori immunoglobulin G antibody
**Oh *et al.***	2010	USA, China	Mix	ESCC	19	8	0	641	195	13	0.675274	N	Age, sex, ethnicity, education, smoking, and alcohol
**Zhang *et al.***	2010	China	Han	ESCC	99	19	2	82	12	1	0.466708	N	Age, and sex
**David *et al.***	2010	Australia	Caucasian	ESCC	128	71	8	842	403	48	0.979478	N	Age, sex, education, BMI, smoking, alcohol, frequency of symptoms of heartburn or reflux, frequency of use of aspirin/NSAIDs past 5 years, and self-reported prior H pylori infection
**Zhao *et al.***	2010	China	Han	ESCC	141	56	5	228	83	6	0.622221	N	Age, and sex
**Umar *et al.***	2013	India	Indian	ESCC	227	62	1	268	42	1	0.631334	Y	Age, sex, alcohol, ethnicity, tumor location, regional lymph node, and environmental exposures of ESCC patients
**Wang *et al.***	2014	China	Kazakh	ESCC	3	26	4	11	25	14	0.979618	Y	Age, sex, and family history
**Cui *et al.***	2015	China	Kazakh	ESCC	150	57	5	140	58	2	0.129934	N	Age, and sex
**David *et al.***	2010	Australia	Caucasian	EAC	157	84	12	842	403	48	0.979478	N	Age, sex, education, BMI, smoking, alcohol, frequency of symptoms of heartburn or reflux, frequency of use of aspirin/NSAIDs past 5 years, and self-reported prior H pylori infection
**Emad *et al.***	2003	USA	Mix	EAC	81	24	3	152	52	6	0.54823	N	Age, sex, BMI, smoking, alcohol, ethnicity, history of reflux symptoms, history of peptic ulcer, family history of cancer, lauren classification, DNA source, and H. pylori immunoglobulin G antibody
**Oh *et al.***	2010	USA, China	Mix	EAC	52	7	0	641	195	13	0.675274	N	Age, sex, ethnicity, education, smoking, and alcohol

a ESCC: esophageal squamous cell carcinoma, EAC: esophageal adenocarcinoma.

b HWE, Hardy-Weinberg equilibrium, P>0.05 indicates that the participants in control group met the HWE.

c “Y” indicates an association between TNF-α-308 G/A polymorphism and risk of ESCC/EAC, “N” indicates no association between this SNP and ESCC/EAC.

### Meta-analysis of crude and adjusted data

**Figure 2:**
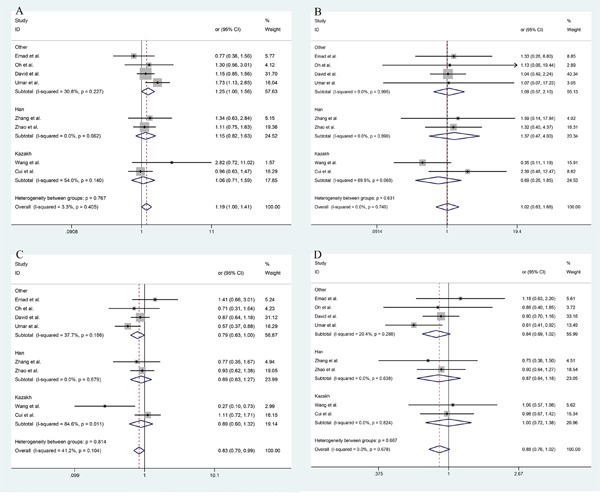
The forest plot of different model and allele frequency (ESCC group) **A.** dominant model; **B.** recessive model; **C.** over-dominant model; **D.** allele frequency.

**Figure 3:**
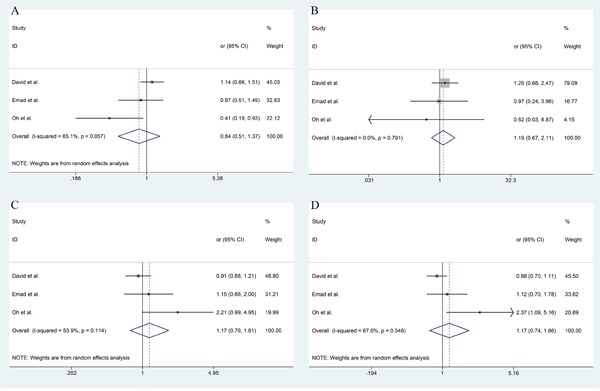
The forest plot of different model and allele frequency (EAC group) **A.** dominant model; **B.** recessive model; **C.** over-dominant model; **D.** allele frequency.

Among these 11 studies, all of the crude and adjusted data are available. The forest plots of different models and allele frequency are shown in Figure 2 (ESCC group) and Figure 3 (EAC group). The I^2^ value of heterogeneities remain in a low level for both crude and adjusted data. This indicates that the studies we included were homogeneous. In ESCC group, dominant model (GA+AA vs GG): OR_adj_=1.19 (1.00, 1.41), recessive model (AA vs GG+GA): OR_adj_=1.02 (0.63, 1.61), over-dominant model (GG vs AA): OR_adj_=0.83 (0.70, 0.99), and allele frequency (G vs A): OR_adj_=0.88 (0.76, 1.02). In EAC group, dominant model (GA+AA vs GG): OR_adj_=0.84 (0.51, 1.37), recessive model (AA vs GG+GA): OR_adj_=1.19 (0.67, 2.11), over-dominant model (GG vs AA): OR_adj_=1.17 (0.76, 1.81), and allele frequency (G vs A): OR_adj_=1.17 (0.74, 1.86). The detailed overall and stratified meta-analyses using crude and adjusted data are shown in Table 2. Meanwhile, the association between TNF-α-308 G/A polymorphism and other cancers is shown in Table 3.

**Table 2:** Meta-analysis of the TNF-α-308 G>A polymorphism on ESCC and EAC group

Group	Comparison	Population	OR (Crude)	OR (Adjusted)	95%CI	Test of association			Model	Heterogeneity I^2^
						*P*	*P*(BON)	*P*(FDR)		
ESCC	GA+AA vs GG	Overall	1.19	1.19	1.00-1.41	0.046	0.160	0.092	F	3.3%
		Han	1.15	1.15	0.82-1.63	0.662	1	0.662	F	0.0%
		Kazakh*	1.06	1.06	1.06-0.71	0.780	-	-	F	54.0%
		Kazakh	1.35	1.35	0.51-3.56	0.550	1	0.994	R	54.0%
		Other	1.25	1.25	1.00-1.56	0.227	0.454	0.302	F	30.8%
	AA vs GG+GA	Overall	1.00	1.02	0.63-1.66	0.929	0.929	0.929	F	0.0%
		Han	1.37	1.37	0.47-4.00	0.569	1	0.662	F	0.0%
		Kazakh*	0.70	0.69	0.26-1.85	0.465	-	-	F	69.9%
		Kazakh	0.85	0.85	0.13-5.44	0.860	1	0.994	R	69.9%
		Other	1.09	1.09	0.57-2.10	0.794	0.794	0.794	F	0.0%
	GG+AA vs GA	Overall	0.83	0.83	0.70-0.99	0.040	0.160	0.092	F	41.2%
		Han	0.89	0.89	0.63-1.27	0.520	1	0.662	F	0.0%
		Kazakh*	0.87	0.89	0.60-1.32	0.564	-	-	F	84.6%
		Kazakh	0.59	0.59	0.15-2.35	0.453	1	0.994	R	84.6%
		Other	0.80	0.79	0.63-1.00	0.049	0.196	0.166	F	37.7%
	G vs A	Overall	0.88	0.88	0.76-1.02	0.085	0.170	0.113	F	0.0%
		Han	0.87	0.87	0.64-1.18	0.377	1	0.662	F	0.0%
		Kazakh	1.00	1.00	0.72-1.02	0.994	1	0.994	F	0.0%
		Other	0.84	0.84	0.69-1.02	0.083	0.249	0.166	F	20.4%
EAC	GA+AA vs GG	Mix	0.89	0.84	0.51-1.37	0.952	1	0.952	R	65.1%
	AA vs GG+GA	Mix	1.16	1.19	0.67-2.11	0.563	1	0.952	F	0.0%
	GG+AA vs GA	Mix	1.05	1.17	0.76-1.81	0.480	1	0.952	R	53.9%
	G vs A	Mix	1.02	1.17	0.74-1.86	0.879	1	0.952	R	67.0%

**OR**, odds ratio; **CI**, confidence intervals; **R**, random effects model; **F**, fixed effects model; P(FDR) *p* value from Benjamini-Hochberg method; P(BON) *p* value in stepdown Bonferroni testing;

T; the values marked with a * represent the results of the alternative approach (fixed effects despite heterogeneity)

**Table 3:** The association between TNF--α-308 G/A polymorphism and other cancers

Study	Cancer Type	Year	Ethnicity	Association^a^
Ming-Hsui Tsai *et al.*	Nasopharyngeal carcinoma	2002	Taiwanese	N
Veljko Flego *et al.*	Lung Cancer	2009	Croatian	N
Carola Seifart *et al.*	Lung Cancer	2005	Caucasian	N
M. M. Stankovic *et al.*	Lung Cancer	2008	Not mentioned	Y
Elvira Garza-González *et al.*	Gastric carcinoma	2005	Mexican	N
Ming-Shiang WU *et al.*	Gastric carcinoma	2004	Chinese	N
Chun Li *et al.*	Gastric carcinoma	2005	Chinese	N
Ja Young Lee *et al.*	Gastric carcinoma	2005	Korean	N
T. Yu *et al.*	Gastric cancer	2014	Chinese Han	Y
Mitsushige Sugimoto *et al.*	Gastric cancer	2007	Japanese	Y
Josecarlos Machado *et al.*	Gastric cancer	2003	Portuguese	Y
Gareth J. Morgan *et al.*	Myeloma	2005	Englishman	N
Lone Skov *et al.*	Basal cell carcinoma	2003	Caucasian	N
Vandana A Govan *et al.*	Cervical cancer	2006	South African	N
Ning Wang *et al.*	Cervical cancer	2012	Chinese	N
Andrzej Roszak *et al.*	Cervical cancer	2015	Polish	Y
Angela DeMichele *et al.*	Breast cancer	2003	American	N
K. C. Smith *et al.*	Breast cancer	2004	Englishman	N
Prithvi Kumar Singh *et al.*	Oral squamous cell carcinoma	2015	Indian	N
Chung-Ji Liu *et al.*	Oral squamous cell carcinoma	2005	Taiwanese	Y
Chuen-Ming Shih *et al.*	Oral cancer	2005	Chinese	Y
Christos Yapijakis *et al.*	Oral cancer	2009	Greek and German	Y
Nega Berhane *et al.*	Prostate cancer	2012	Indian	Y
Bilkay Bas xtürk *et al.*	Renal cell carcinoma	2005	Not mentioned	Y
PILDU JEONG *et al.*	Bladder cancer	2004	Korean	Y
Kathrin Seidemann *et al.*	Non-Hodgkin's Lymphoma	2005	Austrian, German and Swiss	Y
Ho SY *et al.*	Hepatocellular carcinoma	2004	Taiwanese	Y
Penka N. Nikolova *et al.*	Malignant melanoma	2007	Caucasian	Y

a “Y” indicates an association between TNF-α-308 G/A polymorphism and cancer risk, “N” indicates no association between this SNP and cancer risk.

### Sensitivity analysis

**Figure 4:**
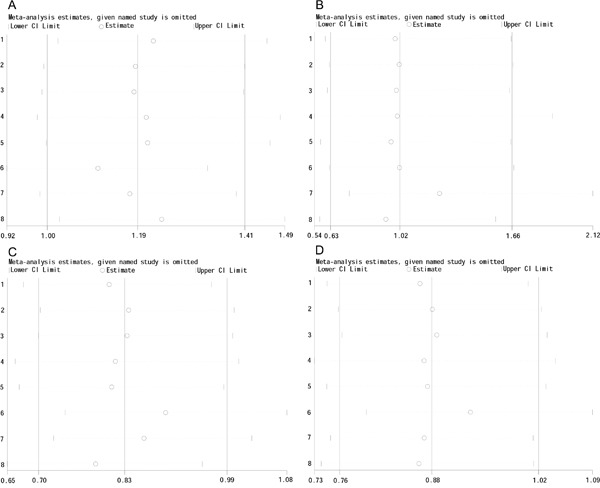
The sensitivity analysis in ESCC group **A.** dominant model; **B.** recessive model; **C.** over-dominant model; **D.** allele frequency.

The stability of the study was detected by “metaninf” module in STATA version 14.0 (STATA Corporation, College Station, TX, USA). The sensitivity analysis indicates that the results was stable. Because of the limited number of studies in EAC group, the analysis was only performed in ESCC group. In ESCC group, the results of sensitivity analysis are shown in Figure 4.

### Assessment of publication bias

**Figure 5:**
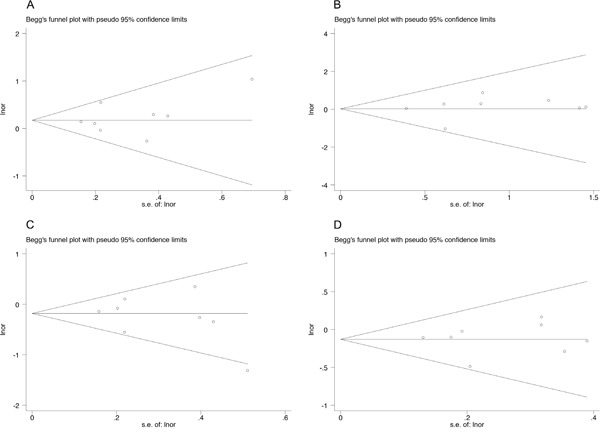
The funnel plot of different model and allele frequency (ESCC group) **A.** dominant model; **B.** recessive model; **C.** over-dominant model; **D.** allele frequency.

In EAC group, we did not assess the publication bias due to the limited number of studies. In ESCC group, the results did not show publication bias, and funnel plots are shown in Figure 5.

## DISCUSSION

Our meta-analysis, which included 1855 cases and 3762 controls, assessed the association between rs1800629 and the risk of EC. Our results indicate that rs1800629 is not significantly associated with an increase in the risk of EC. To achieve greater accuracy, pooled OR with 95% CI were calculated from the selected case–control studies based on both crude and adjusted data. Our results did not change in sensitivity analyses.

Cancer is a multifactor disease that can be caused by genetics, environment, lifestyle, and ethnicity. The frequencies of genetic polymorphisms often vary among ethnic groups. For the TNF-α-308 G/A polymorphism, the prevalence is 17%, 8%, 2%, and 6% in European, Chinese Han, Japanese, and African ancestry groups, respectively, in southwestern USA populations (http://hapmap.ncbi.nlm.nih.gov). A previous study has shown that ethnic-specific differences were evident in the association of CASP8-625 6N del and D302H polymorphisms with prostate cancer risk in east Asian and Indian populations [25]. Liu et al. found that the risk of cardiovascular and end-stage renal diseases in native Asian subjects with type 2 diabetes vary substantially among different ethnic groups (Chinese, Malay, and Indian) [26]. Chu et al. found that the association of a genetic polymorphism with the risk of coronary artery disease varied among different ethnic groups (Chinese, Malay, and Indian) [27]. It is possible that this SNP is associated with different degrees of EC risk in different regions and under diverse environmental conditions.

The TNF-α-308 G/A polymorphism (rs1800629) is located in the promoter region and is associated with increased TNF-α production [28]. TNF is a cytokine involved in systemic inflammation, and it is produced chiefly by immune cells (especially macrophages, dendritic cells, lymphocytes, and mast cells) as a transmembrane protein [29]. Deans et al. found the AA TNFα 308 genotype to be related to an adverse prognosis of gastro-oesophageal cancer. In contrast to the IL-6 and IL-10 genotypes, there is no association between TNFα polymorphisms and systemic inflammation [30]. It is therefore less likely that the reduced survival associated with this cytokine is related entirely to the generation of an inflammatory response.

The AA genotype of TNF-α-308 can bind to two receptors in cellular responses: TNFR1 (TNF receptor type 1) and TNFR2 (TNF receptor type 2) [31]. Through these two distinct receptors, TNF can activate pathways leading to diverse cellular effects: cell survival, activation, differentiation, proliferation, and cell death. TNF can induce the expression of transforming growth factor alpha (TGF α) and epidermal growth factor receptor (EGFR) in cancer cells [32–34]. This means TNF may have a role in the proliferation of cancer cells.

The TNF-α-308 G/A polymorphism is an interesting SNP site. It has been reported to be associated with Cervical cancer, Gastric cancer, Oral cancer, Prostate cancer, Renal cell carcinoma, Bladder cancer, Hepatocellular carcinoma, Malignant melanoma and Non-Hodgkin's Lymphoma [35–48]. However, no significant association was found for Nasopharyngeal carcinoma, Lung cancer, Gastric carcinoma, Myeloma, Basal cell carcinoma, Cervical cancer, Breast cancer and Oral squamous cell carcinoma [49–62]. The association between this polymorphism and these cancer risk can be found in Table 3. Among inflammatory cytokines, TNF α is not the only one that is not significantly associated with cancer risk. Other polymorphisms, such as the interleukin 10-1082 G/A polymorphism, have no association with cervical cancer [63], esophageal squamous cell carcinoma, or gastric cardiac adenocarcinoma risk [64]. Thus, there is no obvious association between the TNF-α-308 G/A polymorphism and cancer risk. The controversial association between TNF-α-308 G/A polymorphism and cancer still needs to be confirmed.

With the development of high-throughput sequencing, the need for knowledge to assist precision medicine is increasing. In the era of precision medicine, a negative result has important implications. For a specific disease, a negative result can help us filter out the noise variant from all of the sequencing data.

Several limitations should be taken into consideration. First, all included studies were published in English or Chinese. Studies published in other languages were not included, and the annual incidence rates of EC have been decreasing in recently years [65]. In other words, the number of patients with esophageal cancer is decreasing. Meanwhile, there might have unpublished negative studies that we cannot include in our analysis. Therefore, the conclusion of the null association between rs1800629 and EC might be weakened by the small sample size and publication bias. Second, heterogeneity was obvious in some groups in the sub-group analysis. However, the results did not change in a sensitivity analysis. Finally, this polymorphism may have a combinational effect of a SNP-SNP interaction on the EC risk; however, we cannot estimate this from the data reported by the included original studies.

In conclusion, our meta-analysis shows that TNF-α-308 G>A (rs1800629) is not significantly associated with an increased esophageal cancer risk. For genetic association studies, negative results of meta-analysis have a high level of evidence, and thus, our results have special importance in the era of sequencing-based precision medicine. However, given the limitations of this meta-analysis, it is necessary to perform further experiments with more participants to confirm our conclusion.

## MATERIALS AND METHODS

### Search strategy

PubMed, Embase, Web of Science and CNKI database were searched by using the keywords “TNF α [MeSH]” and “esophageal cancer [MeSH]” for all studies published up to August 24, 2016. Studies that investigate the association between rs1800629 and risk of esophageal cancer were included in this meta-analysis.

### Criterion for study selection

The following inclusion criteria were set and reviewed by two independent investigators: (1) an independent case-control design, (2) evaluating the association between rs1800629 and risk of EC, (3) providing the number of genotypes in case-control groups for calculating odds ratio (OR) with 95% confidential intervals (CIs), and (4) genotypes of participants in control groups satisfy Hardy-Weinberg equilibrium (HWE).

### Data extraction

Two independent reviewers collected data from studies included in the meta-analysis. The following data were included: first author, publication date, country, ethnicity of the study participants, type of esophageal cancer, number of genotypes in case-control groups and the association between rs1800629 and EC. In addition, *P* value of Hardy-Weinberg equilibrium in control group was calculated to evaluate whether the sample in control groups comes from the same Mendel groups where allele and genotype frequencies in a population will remain constant from generation to generation in the absence of other evolutionary influences.

### Statistical analyses

The association between the rs1800629 and EC risk was measured by Stata 12.0 to calculate the odds ratio (OR) with 95% confidence intervals (CIs). The Chi-squared test and Higgins's (I^2^) test were used to assess heterogeneity. We used the fixed effects model to combine the data if I^2^ < 50% or the *P*-value of heterogeneity was > 0.10. Otherwise, the random effects model was chosen. Pooled OR with 95% CI were calculated for case–control studies based on both crude and adjusted data for the selected studies. For the crude data analysis, we used the number of people with or without esophageal cancer in the case and control group. For the analysis of the adjusted data, we extracted the OR with 95% CI that had been adjusted for various potential confounders [66]. The Z-test was used to determine the significance of the overall OR, and *P*<0.05 was considered statistically significant. In order to adjust for multiple comparisons, we used the Benjamini-Hochberg (BH) method and stepdown Bonferroni method, which control for false discovery rate (FDR) and familywise error rate (FWE), respectively. The sensitivity analysis was measured by “metaninf” module. The Begg's rank correlation test and Egger's linear regression test were used to assess the publication bias, and *P*<0.05 was considered statistically significant. All statistical analyses were conducted by STATA version 14.0 (STATA Corporation, College Station, TX, USA) and R package version 3.2.3.
